# Long-Term Clinical Outcomes After Physiology-Guided vs Angiography-Guided Coronary Artery Bypass Grafting: From the FARGO Trial

**DOI:** 10.1016/j.jacadv.2026.103332

**Published:** 2026-09-22

**Authors:** Jens Trøan, Anne Langhoff Thuesen, Evald Høj Christiansen, Ashkan Eftekhari, Ole Havndrup, Ivy Susanne Modrau, Lars Peter Riber, Jan Jesper Andreasen, Svend Eggert Jensen, Joachim Dahl Steensbjerre, Julie Juul Siig, Yusuf Abdi Isse, Anders Junker, Poul Erik Mortensen, William F. Fearon, Lisette Okkels Jensen

**Affiliations:** a Department of Cardiology, Odense University Hospital, Odense, Denmark; b Department of Cardiothoracic Surgery, Odense University Hospital, Odense, Denmark; c Department of Cardiology, Aarhus University Hospital, Aarhus, Denmark; d Department of Cardiology, Aalborg University Hospital, Aalborg, Denmark; e Department of Cardiology, Zealand University Hospital, Roskilde, Denmark; f Department of Cardiothoracic and Vascular Surgery, Aarhus University Hospital, Aarhus, Denmark; g Department of Clinical Medicine, Aarhus University, Aarhus, Denmark; h Department of Cardiothoracic Surgery, Aalborg University Hospital, Aalborg, Denmark; i DaCHE, Department of Public Health, University of Southern Denmark, Odense, Denmark; j Department of Medicine, Stanford University, Stanford, California, USA; k Stanford Cardiovascular Institute, Stanford University, Stanford, California, USA; l VA Palo Alto Health Care System, Palo Alto, California, USA

**Keywords:** angiography, clinical outcomes, coronary artery bypass grafting, fractional flow reserve

## Abstract

**Background:**

Fractional flow reserve (FFR)-guided coronary artery bypass grafting (CABG) may reduce unnecessary grafts and improve outcomes. However, in the FARGO (Fractional Flow Reserve vs Angiography Randomization for Graft Optimization) trial, FFR was reduced significantly after 6 months in deferred lesions.

**Objectives:**

The purpose of this study was to evaluate long-term clinical outcomes after FFR-guided CABG compared to angiography-guided CABG.

**Methods:**

In this multicenter randomized clinical trial, 100 patients were randomized to FFR-guided (n = 50) or angiography-guided CABG (n = 50). Patients underwent a 6-month follow-up coronary angiography with FFR in deferred lesions. In this extended long-term follow-up revascularization, myocardial infarction, stroke, death, and major adverse cardiac and cerebrovascular events (MACCE), a composite of the individual endpoints, were assessed.

**Results:**

After a median follow-up of 8.8 years (IQR: 8.3, 9.3), patients in the FFR-guided group had significantly more MACCE compared to the angiography-guided group, (26 [52%] vs 16 [32%], log-rank test *P* = 0.03). This difference was driven by significantly more long-term revascularization, FFR-guided CABG 32% vs angiography-guided 12%, log-rank test *P* = 0.01. The revascularization rate was significantly higher from 0 to 1 year (12 [24%] vs 3 [6%], log-rank test *P* = 0.01), whereas beyond 1 year, rates were similar (4 [8%] vs 3 [6%], log-rank test *P* = 0.47). Most patients had stable symptoms, and revascularization was symptom-driven. Myocardial infarction (6 [12%] vs 3 [6%]), death (10 [20%] vs 7 [14%]), and stroke (6 [12%] vs 6 [12%]) did not differ significantly between the groups.

**Conclusions:**

FFR-guided CABG was associated with a significantly higher rate of MACCE after CABG, driven by more revascularization within the first year.

Treatment of coronary artery disease consists of medical therapy and revascularization.^1^^,^^2^ Revascularization can be achieved either by percutaneous coronary intervention (PCI) or by coronary artery bypass grafting (CABG), which improves symptoms and is associated with improved clinical outcomes.^2^ Initial invasive assessment of the extent of coronary artery disease is made visually during coronary angiography (CAG).^2^ However, visual assessment often overestimates the functional severity of a stenosis.^3^ The functional significance of coronary artery stenosis can be evaluated invasively using fractional flow reserve (FFR), where an FFR value of <0.80 is considered significant.^2^^,^^4^, ^5^, ^6^ FFR-guided PCI has shown improvement in symptoms and outcomes compared to angiography-guided PCI while simultaneously reducing the number of treated vessels.^5^^,^^7^ Several studies have shown that deferral of nonsignificant lesions, assessed by FFR, is safe and associated with a low risk of long-term adverse clinical outcomes.^8^, ^9^, ^10^ While the results of FFR-guided PCI have been encouraging, less is known about the role of FFR in guiding CABG. Previous studies have shown FFR-guided CABG to be associated with a reduction in grafted vessels compared to angiography-guided CABG and significantly fewer graft failures in FFR significant vessels.^11^, ^12^, ^13^, ^14^ However, the clinical impact of FFR-guided CABG remains unclear, particularly regarding subsequent revascularization and major adverse cardiac events, limited by the small number of randomized controlled trials and short follow-up duration.^15^^,^^16^

In the FARGO (Fractional Flow Reserve vs Angiography Randomization for Graft Optimization) trial, similar rates of graft failure were observed in the FFR-guided group (16%) compared to the angiography-guided group (12%), in addition to similar improvements in health-related quality of life. However, FFR was significantly reduced in deferred lesions after 6 months,^16^ raising the question of whether FFR-guided deferral of revascularization is safe in CABG. Despite deferred lesion progression, improvement in health-related quality of life was still seen compared with baseline before CABG. Therefore, this extended follow-up of the FARGO trial evaluates the long-term clinical outcomes after FFR-guided CABG and angiography-guided CABG.

## Methods

The FARGO trial (NCT02477371) was a prospective, open-label, multicenter, randomized clinical trial comparing FFR-guided CABG with angiography-guided CABG. The study was conducted at Odense University Hospital, Aarhus University Hospital, and Aalborg University Hospital from June 2014 to December 2016. All patients provided written informed consent to participate, and the study was approved by the Danish Research Ethics Committee (S-20130050) and the Danish Data Protection Agency (14/9063). The primary results have previously been published.^16^

### Patient selection

Patients referred for CABG with stable angina, unstable angina, or non–ST-segment elevation myocardial infarction (MI) were included in the study. Patients were eligible if they had one or more study lesions planned for grafting by the heart team. A study lesion was defined as being a ≥50% visually assessed stenosis of a major epicardial artery >2.5 mm in diameter (proximal reference) that was deemed passable by a pressure wire. Patients were excluded if they were referred for concomitant valve surgery, had prior heart surgery, left main disease with either occlusion or no significant stenosis of the right coronary artery (RCA), single-vessel disease, creatinine ≥150 μmol/L, treatment with dipyridamole, or if they were referred for an emergent operation.

### Randomization

Patients were randomized to FFR-guided CABG or angiography-guided CABG using the web-based TrialPartner randomization system. Patients were allocated in a 1:1 ratio, stratified by the presence of diabetes, sex, and study center using permuted blocks.

### Graft plan

The local heart team created a graft plan for all individual patients based on the CAG. Afterward, patients were randomized to either FFR-guided or angiography-guided CABG. Thereafter, patients underwent another invasive evaluation with FFR measurements of study lesions. In some patients, FFR evaluation was performed before the heart team discussion, and in these patients, the heart team was blinded to the FFR values, and a graft plan was made based solely on the CAG. In the FFR-guided group, study lesions with an FFR ≤0.80 were planned for grafting, while lesions with FFR >0.80 were deferred. In the angiography-guided group, the operating surgeon was blinded to the FFR values, and the initial graft plan of the blinded heart team was followed.

### Invasive diagnostic procedure

The CAG was performed following local hospital guidelines for the assessment of study lesions. Before FFR evaluation all patients received intravenous heparin and intracoronary nitroglycerine. A coronary artery pressure wire (Pressure Wire X, St. Jude Medical) was advanced distally to the study lesion, after equalizing at the guiding catheter tip. Afterward, intravenous adenosine with a dose of 140 μg/kg/min was infused for 2 minutes to ensure maximal hyperemia before the FFR measurement was performed. FFR was defined as the ratio of the mean pressure distal to the study lesion to the aortic pressure measured at the guiding catheter.

### Coronary artery bypass grafting

The procedure was performed according to the final graft plan based on the randomization. The left and right internal mammary arteries (LIMA/RIMA), the radial artery, and saphenous veins were used as graft material. LIMA, RIMA, and radial artery were harvested using an open-technique, while some veins were harvested using either a no-touch or an endoscopic technique. The decision regarding off-pump surgery was left to the operating surgeon. All patients received postoperative and postdischarge care according to local hospital and current clinical guidelines. Medical therapy followed best clinical practice, where all patients without contraindications were given statin and aspirin therapy. In addition, if the indication for surgery was acute coronary syndrome, then patients received ticagrelor 90 mg twice daily for 12 months.

### Follow-up

Patients were invited for follow-up CAG 6 months after the CABG. Graft occlusion was assessed in all patients, whereas patients in the FFR-guided group underwent FFR evaluation of deferred lesions. Care was taken to ensure that FFR measurements were obtained at the same position as at baseline. Clinical follow-up, using the electronic patient record, was conducted in all included patients from the time of CABG to December 31, 2024.

### Outcomes

Long-term clinical outcomes, as defined below, were assessed. All endpoints were obtained using the electronic patient record. Follow-up was not scheduled after the 6-month angiographic control but only clinically driven, based on patients seeking medical care for self-reported angina or equivalent symptoms.

### Definitions

Any new revascularization was defined as any new revascularization either by CABG or PCI after the completion of CABG until the end of follow-up. In patients where a hybrid procedure was planned, the PCI was not considered an any new revascularization event, as it was considered part of the intended treatment strategy.

MI was defined according to the third definition used by the American Heart Association, American College of Cardiology, the European Society of Cardiology, and the World Heart Federation.^17^

Stroke was defined according to the updated definition by the American Heart Association and American Stroke Association.^18^ All patients received the International Classification of Diseases-10th Revision (ICD-10) diagnosis of stroke.

Vital status was obtained based on the electronic patient journal. The cause of death was based on electronic records. If the cause of death could be determined, death was classified as cardiac.

Major adverse cardiac and cerebrovascular events (MACCE) was defined as a composite of death, MI, stroke, or new revascularization. A combined safety endpoint was defined as a composite of death, MI, and stroke.

### Sample size

The original sample size for the FARGO trial was calculated based on the primary endpoint of graft patency at 6-month follow-up. Based on Botman et al,^12^ a graft occlusion rate of 20% in lesions with FFR >0.80 and a graft occlusion rate of 5% in lesions with FFR ≤0.80 were assumed. A minimum sample of 84 patients per group was estimated to provide 80% statistical power using a 2-sided chi-squared test with an alpha of 0.05.

### Statistics

Continuous data were presented as mean ± SD, when normally distributed, and compared using the Student’s *t*-test. Nonparametric tests were used when data were not normally distributed and presented as the median and 25th and 75th quartile ranges (IQR). Categorical data were presented as numbers and frequencies and compared using the chi-square test and Fisher exact test when expected counts were <5. Incidence rates were presented as numbers and frequencies and compared using the log-rank test. Cox proportional hazards regression analyses were used to estimate HRs with 95% CIs. Since randomization was performed in permutated boxes by center stratified by sex and the presence of diabetes, these factors were included as covariates in the model. HRs were presented as crude HR and adjusted HR. The proportional hazard assumption was evaluated using Schoenfeld residuals. A Fine-Gray analysis was conducted as a sensitivity analysis, accounting for the competing risk of death. In addition, sensitivity analyses excluding events detected at the mandatory follow-up were conducted for MACCE and any new revascularization. All analyses were performed using the intention-to-treat principle, and all patients, 100 patients were included according to randomized group allocation, and no patients had missing data. A 2-sided *P* value of <0.05 was considered statistically significant. All statistical analyses were conducted using STATA 17.0 software (StataCorp).

## Results

A total of 100 patients were included and randomized to either FFR-guided CABG or angiography-guided CABG. In 3 patients, FFR evaluation after randomization was not possible because the study lesion could not safely be crossed with a pressure wire due to severe stenosis, resulting in 49 patients in the FFR-guided group and 48 patients in the angiography-guided group (Figure 1). Clinical follow-up was conducted in all 100 included patients with a median follow-up time of 8.8 years (IQR: 8.3, 9.3).

**Figure 1:**
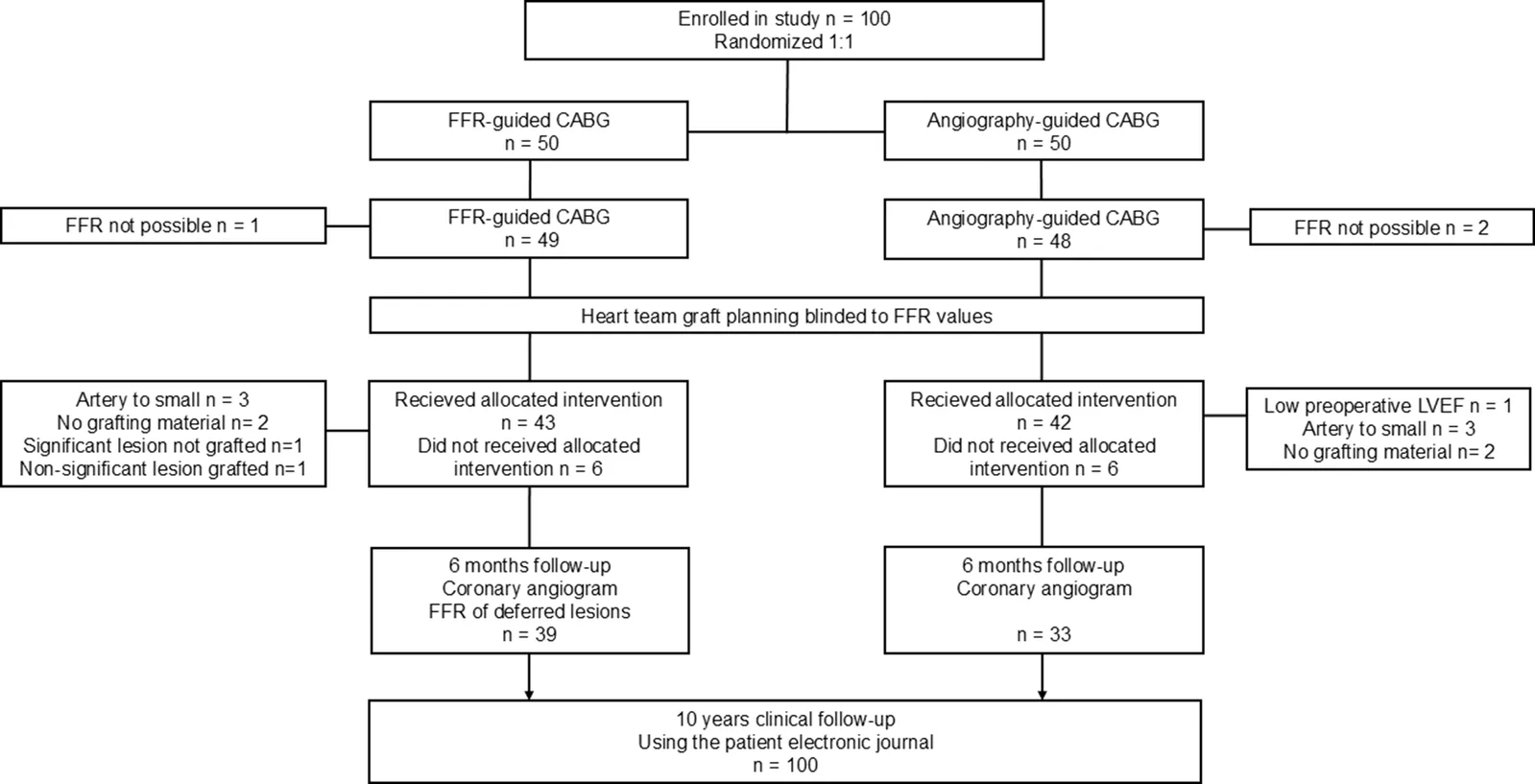
CONSORT Diagram for the FARGO Trial CONSORT diagram for the FARGO (Fractional Flow Reserve vs Angiography Randomization for Graft Optimization) trial. CABG = coronary artery bypass grafting; CONSORT = consolidated standards of reporting trial; FFR = fractional flow reserve; LVEF = left ventricular ejection fraction.

### Patient and procedural characteristics

Patient baseline and procedural characteristics of the FFR-guided group compared to the angiography-guided group are presented in Table 1. There were no differences in baseline characteristics between the 2 groups. In total, 275 arteries were grafted, of which 102 (37%) grafts were arterial. In the FFR-guided group, 31 study lesions were deferred from grafting. FFR-guided CABG resulted in significantly fewer grafts per patient compared to the angiography-guided group, 2.5 ± 0.9 vs 3.0 ± 0.9, *P* = 0.002. The patients were grafted according to the graft plan in 43 (88%) patients in the FFR-guided group and 42 (88%) patients in the angiography-guided group. Due to poor target vessels, 4 patients (FFR-guided: 3 and angiography-guided: 1) were treated with a hybrid revascularization strategy.

**Table 1:** Patient and Procedural Characteristics

	FFR-Guided Group (n = 50)	Angiography-Guided (n = 50)	*P* Value
Age, y, mean (SD)	66.5 (6.4)	65.5 (8.8)	0.53
Male, n (%)	44 (88%)	45 (90%)	0.75
Hypertension, n (%)	34 (68%)	33 (66%)	0.83
Hyperlipidemia, n (%)	43 (86%)	38 (76%)	0.20
Family history of IHD, n (%)	26 (52%)	27 (54%)	0.60
Diabetes mellitus, n (%)	11 (22%)	11 (22%)	1.00
Current smoking, n (%)	13 (27%)	8 (16%)	0.20
Prior PCI, n (%)	14 (28%)	10 (20%)	0.35
Prior myocardial infarction, n (%)	11 (22%)	12 (24%)	0.81
Left ventricular ejection fraction, n (%)			0.67
≥55%	35 (70%)	33 (66%)	
<55%	15 (30%)	17 (34%)	
Euroscore I (%), mean (SD)	2.5 (1.8)	2.9 (2.8)	0.33
Euroscore II (%), mean (SD)	1.1 (0.6)	1.2 (1.1)	0.36
On-pump surgery, n (%)	45 (90%)	48 (96%)	0.24
Indication for surgery, n (%)			0.06
Stable angina	43 (86%)	34 (68%)	
Unstable angina	1 (2%)	6 (12%)	
Non–ST-segment elevation myocardial infarction	6 (12%)	10 (20%)	
Vessel disease, n (%)			0.77
1	1 (2%)	1 (2%)	
2	10 (20%)	13 (26%)	
3	39 (78%)	36 (72%)	
Number of grafts, mean (SD)	2.5 (0.9)	3.0 (0.9)	0.002
Grafted vessel characteristics			
Total grafts, n	124	151	0.76
Left anterior descending artery, n (%)	48 (38.7%)	50 (33.1%)	
Diagonal, n (%)	10 (8.1%)	11 (7.3%)	
Circumflex artery, n (%)	38 (30.6%)	53 (35.1%)	
Right coronary artery, n (%)	28 (22.6%)	37 (24.5%)	

FFR = fractional flow reserve; IHD = ischemic heart disease; PCI = percutaneous coronary intervention.

### Angiographic follow-up

As reported in the main FARGO trial publication,^16^ FFR in deferred vessels decreased significantly from before CABG to the 6-month control, baseline median FFR 0.88 (IQR: 0.84, 0.94) vs 6-month median FFR 0.83 (IQR: 0.78, 0.89), *P* = 0.01 (Figure 2).

**Figure 2:**
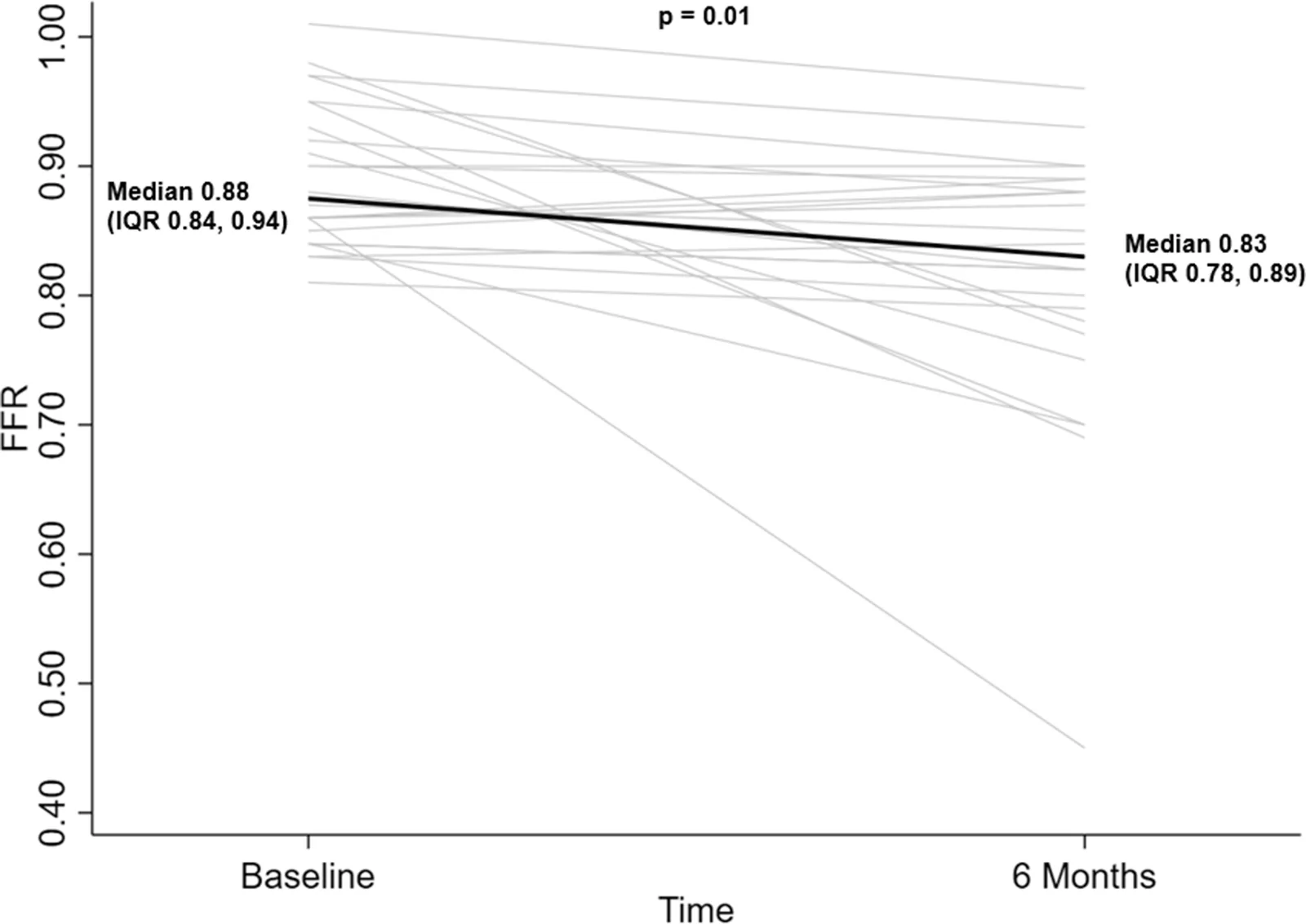
Change in Fractional Flow Reserve From Baseline to 6 Months Follow-Up Spaghetti plot of the fractional flow reserve measurements at baseline and at 6-month control in deferred lesions. Gray lines connecting measurements in the same patients, while the black lines connecting the two medians. Abbreviation as in Figure 1.

### Clinical follow-up

MACCE was significantly higher in the FFR-guided group compared to the angiography-guided group, 26 (52%) vs 16 (32%), HR: 2.01; 95% CI: 1.08-3.75, *P* = 0.03, log-rank test *P* = 0.03 (Figure 3A). The difference in MACCE was mainly driven by significantly more revascularization in the FFR-guided group compared to the angiography-guided group, 16 (32%) vs 6 (12%), HR: 3.14; 95% CI: 1.23-8.02, *P* value = 0.017, log-rank test *P* = 0.01 (Figure 3B). The increased revascularization rate was driven by a significantly higher revascularization within the first year, FFR-guided group 12 (24%) vs angiography-guided group 3 (6%), HR: 4.50; 95% CI: 1.27-15.96, *P* = 0.02, log-rank test *P* = 0.01. Whereas there was no difference from 1 year to the end of follow-up in the rate of new revascularization, FFR-guided: 4 (8%) vs angiography-guided: 3 (6%), HR: 1.72; 95% CI: 0.38-7.69, *P* = 0.48, log-rank *P* = 0.47. The adjusted HR yielded similar results (Table 2). The reasons for revascularization are presented in Table 3. In 5 patients in the FFR-guided group, the revascularization was due to deferred lesion progression and symptoms of ischemia (FFR 0.91 [IQR: 0.86, 0.93] vs 6-month median FFR 0.70 [IQR: 0.69, 0.70], *P* = 0.06). Of the 5 FFR deferred lesions that underwent revascularization, 4 were in the RCA and 1 in the left circumflex artery. In 1 patient in the FFR-guided group, a functionally significant RCA stenosis was left untreated during the CABG, and the patient later developed angina and subsequently underwent revascularization.

**Figure 3:**
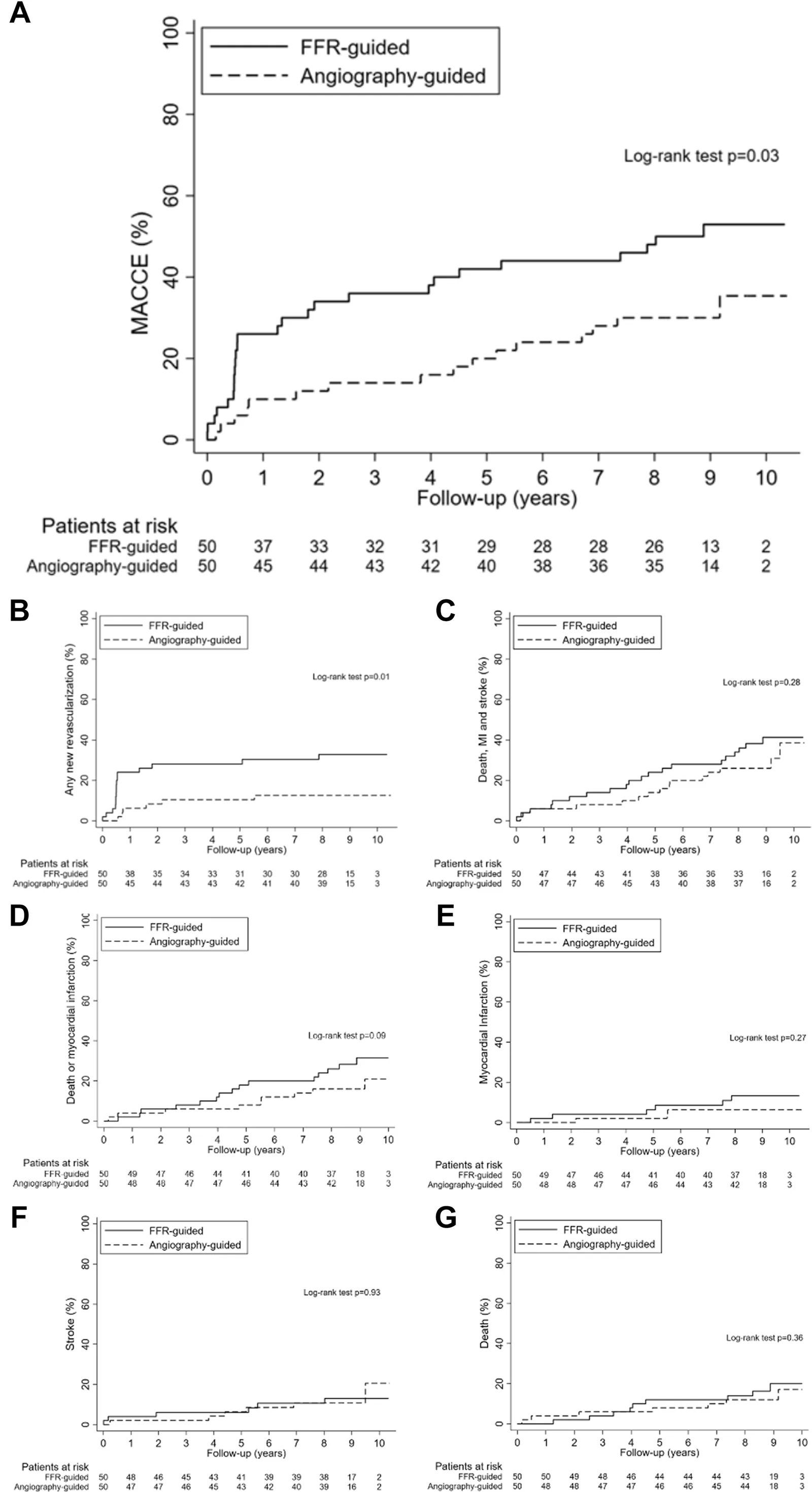
Kaplan-Meier Curve for Fractional Flow Reserve-Guided Coronary Bypass Grafting vs Angiography-Guided Coronary Bypass Grafting Time-to-event curve for (A) major adverse cardiac and cerebrovascular events, (B) any new revascularization, (C) combined safety endpoint of death, myocardial infarction, and stroke, (D) death or myocardial infarction, (E) myocardial infarction, (F) stroke, and (G) death for patients in the fractional flow reserve-guided group compared to the angiography-guided group. MI = myocardial infarction; MACCE = major adverse cardiac and cerebrovascular events; other abbreviation as in Figure 1.

**Table 2:** Long-Term Clinical Outcomes After Coronary Artery Bypass Grafting

	FFR-Guided Group (n = 50)	Angiography-Guided (n = 50)	Crude HR (95% CI)	Adjusted HR (95% CI)	*P* Value^a^
Major adverse cardiac and cerebrovascular events, n (%)					
0 y to end of follow-up	26 (52%)	16 (32%)	2.01 (1.08-3.75)	2.11 (1.12-3.97)	0.03
0 to 1 y	13 (26%)	5 (10%)	2.90 (1.03-8.13)	2.91 (1.02-8.27)	0.03
1 y to end of follow-up	13 (26%)	11 (22%)	1.57 (0.70-3.52)	1.62 (0.72-3.68)	0.26
Any new revascularization, n (%)					
0 y to end of follow-up	16 (32%)	6 (12%)	3.14 (1.23-8.02)	3.43 (1.32-8.94)	0.01
0 to 1 y	12 (24%)	3 (6%)	4.50 (1.27-15.96)	5.03 (1.38-18.42)	0.01
1 y to end of follow-up	4 (8%)	3 (6%)	1.72 (0.38-7.69)	1.93 (0.41-9.02)	0.47
Death, MI and stroke, n (%)	20 (40%)	15 (30%)	1.45 (0.74-2.83)	1.42 (0.73-2.78)	0.28
Death or MI, n (%)	16 (32%)	9 (18%)	2.00 (0.88-4.55)	1.96 (0.86-4.46)	0.09
MI, n (%)	6 (12%)	3 (6%)	2.14 (0.54-8.57)	2.03 (0.50-8.19)	0.27
Stroke, n (%)	6 (12%)	6 (12%)	1.05 (0.34-3.26)	1.09 (0.35-3.38)	0.93
All-cause mortality, n (%)	10 (20%)	7 (14%)	1.57 (0.59-4.17)	1.56 (0.58-4.17)	0.36
Cardiac death, n (%)	3 (6%)	3 (6%)	1.01 (0.20-4.99)	0.91 (0.18-4.64)	0.99

MI = myocardial infarction; other abbreviation as in Table 1.

a Numbers are compared using log-rank test. Cox proportional hazards regression analyses were used to estimate HRs with 95% CIs adjusted for including center, sex and presence of diabetes.

**Table 3:** Indication for Long-Term New Revascularization After Coronary Bypass Grafting

	FFR-Guided Group (n = 16)	Angiography-Guided (n = 6)	Total (N = 22)
Deferred lesion progression, n (%)	5 (31%)	0 (0%)	5 (23%)
Graft failure, n (%)	7 (44%)	5 (83%)	12 (55%)
Progression of atherosclerosis/de novo lesions	3 (19%)	1 (17%)	4 (18%)
Others	1 (6%)	0 (0%)	1 (4%)

Abbreviation as in Table 1.

Among the 31 deferred lesions in the FFR-guided group, 5 (16%) were revascularized during the clinical follow-up period, while 4 (12.9%) were revascularized within the first year after CABG. All 4 revascularizations were performed in relation to the 6-month angiographic control. However, all lesion revascularizations of deferred lesions were ischemia-driven, based on both patients’ symptoms and a functionally significant FFR value.

There was no difference in the rate of MI in the FFR-guided group compared to the angiography-guided group, MI (6 [12%]) vs (3 [6%]), HR: 2.14; 95% CI: 0.54-8.57, *P* = 0.28, log-rank test *P* = 0.27 (Figure 3E). Furthermore, there was no difference in stroke between the 2 groups (FFR-guided 6 [12%]) vs angiography-guided (6 [12%]), HR: 1.05; 95% CI: 0.34-3.26, *P* = 0.93, log-rank test = 0.93) (Figure 3F). In addition, all-cause mortality was similar in the FFR-guided group and the angiography-guided group (10 [20%] vs 7 [14%], HR: 1.57; 95% CI: 0.59-4.17, *P* = 0.36, log-rank test *P* = 0.36) (Figure 3G). There was no difference in the combined safety endpoint of death, MI, and stroke (FFR-guided: 20 [40%] vs angiography-guided: 15 [30%], HR: 1.45; 95% CI: 0.74 to 2.83, *P* = 0.28, log-rank test *P* = 0.28) (Figure 3C), nor in a combined endpoint of death or MI (FFR-guided: 16 [32%] vs angiography-guided: 9 [18%], HR: 2.00; 95% CI: 0.88-4.55, *P* value = 0.10, log-rank test *P* = 0.09) (Figure 3D). Similar results were seen in all adjusted HRs (Table 2).

When excluding events found during the mandatory 6-month follow-up, a numerically higher rate of MACCE (FFR-guided: 22 [44%] vs angiography-guided: 16 [32%]) and any new revascularization (FFR-guided: 8 [16%] vs angiography-guided: 5 [10%]) was seen in the FFR-guided group compared to the angiography-guided group. However, the results were no longer statistically significant for MACCE (FFR-guided: HR: 1.49; 95% CI: 0.78-2.84, *P* = 0.23) or any new revascularization (FFR-guided vs angiography-guided: HR: 1.67; 95% CI: 0.55-5.11: *P* = 0.37).

The proportional hazards assumption was met in all variables (Schoenfeld residual test *P* > 0.05), apart from the rejection of the proportional assumption only in the revascularization rate from 0 to 1 year (*P* = 0.0368). Therefore, the reported HR for any new revascularization from 0 to 1 year should be interpreted as a weighted average of the relative hazard over the first year of follow-up, influenced by the timing of events censoring, rather than a constant treatment effect throughout the follow-up period. Furthermore, Fine-Gray analyses of all endpoints not including death showed similar results to the primary analysis (Supplemental Table 1).

## Discussion

The extended follow-up of the FARGO trial compared long-term clinical outcomes of FFR-guided CABG compared to angiography-guided CABG and showed that FFR-guided CABG was associated with an increased rate of any new revascularization after CABG compared to angiography-guided CABG (Central Illustration). The increased revascularization rate appeared to be related to the progression of deferred lesions, graft failure, and de novo lesions. Finally, FFR-guided CABG and angiography-guided CABG had similar rates of MI, stroke, and all-cause mortality.

**Central Illustration:**
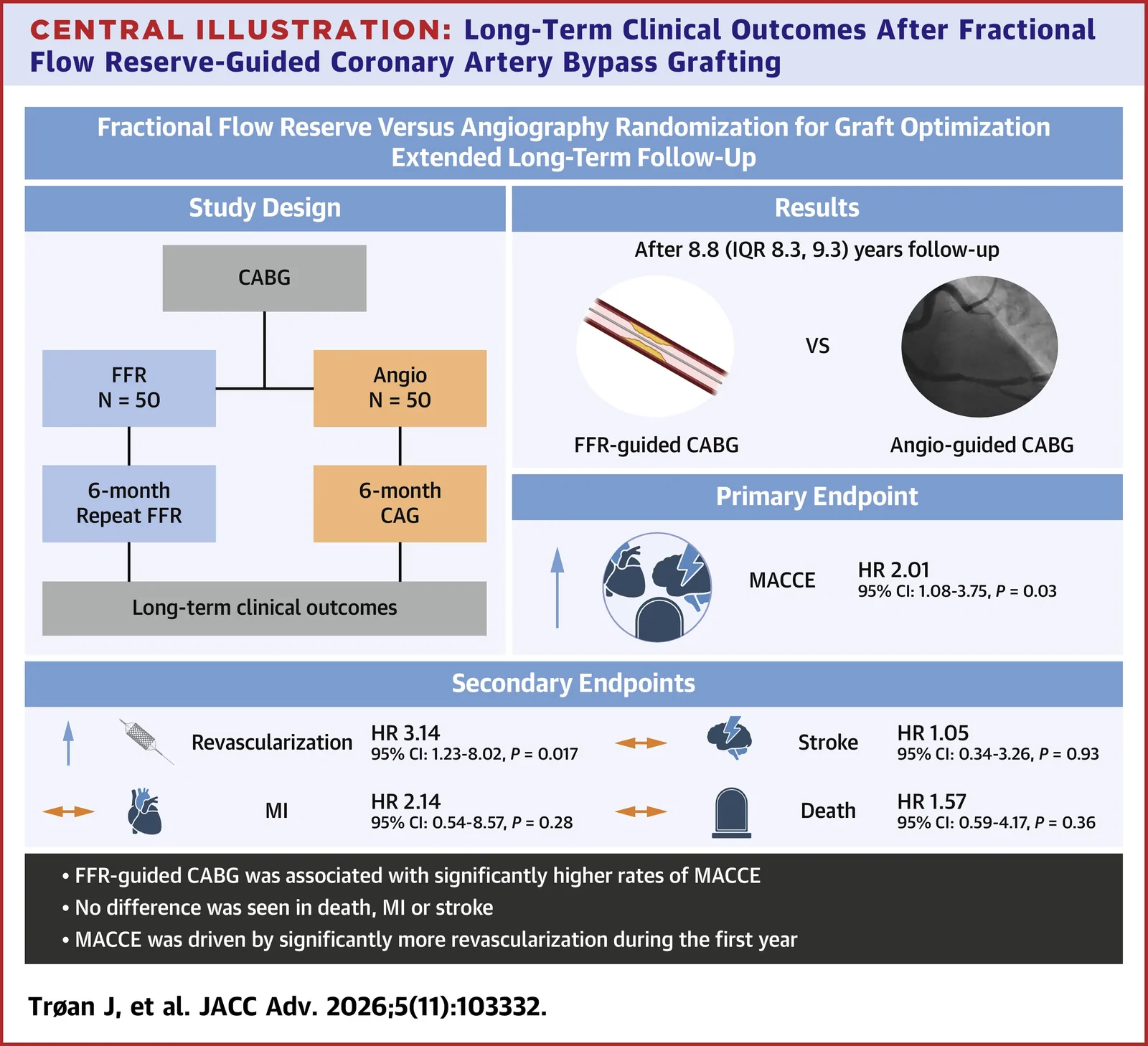
Long-Term Clinical Outcomes After Fractional Flow Reserve-Guided Coronary Artery Bypass Grafting In this extended follow-up of the multicenter, clinical, randomized, FARGO (Fractional Flow Reserve vs Angiography Randomization For Graft OptimizationFARGO) trial, long-term clinical outcomes were assessed. Patients randomized to fractional flow reserve-guided coronary artery bypass grafting (coronary artery bypass grafting compared to angiography-guided coronary artery bypass grafting had significantly more major adverse cardiac and cerebrovascular events, driven by significantly more new revascularization. Created in Biorender. Abbreviations as in Figures 1 and 3.

The results of the present study suggest that FFR-guided CABG may not provide a benefit on the revascularization rate and possibly associated with reduced efficacy. Two previous studies have reported on revascularization after physiology-guided CABG.^13^^,^^19^ The GRAFFITI (GRAft patency after FFR-guided versus angiography-guIded CABG) trial is the only other randomized trial for FFR-guided vs angiography-guided CABG.^13^ They found no difference in the target-vessel revascularization (TVR) rate at 12 months of follow-up between the 2 groups. The TVR rate was also substantially lower in the GRAFFITI trial compared to the revascularization rate observed in the present study, with only 3.4% after 12 months, compared to 18% in the present study. Patients in the GRAFFITI trial were more frequently female, had less prior PCI, and were more often referred due to stable angina, indicating a lower overall risk of repeat revascularization compared to the present study. In addition, the median FFR in the FFR-guided group was 0.70 in the GRAFFITI trial, while the median FFR was 0.78 in the present study, suggesting a greater stenosis severity. Greater stenosis severity assessed by FFR is associated with more benefit from revascularization on major adverse cardiovascular events,^20^ and may explain some of the difference between the 2 studies. However, in the GRAFFITI trial, the angiographic follow-up was conducted after 1 year without mandated FFR evaluation and was mainly conducted by coronary computed tomography angiography. This difference could explain the lower revascularization rate in the GRAFFITI trial, as mandated invasive follow-up with FFR might have given rise to study-induced revascularization in the present study. In the prospective nonrandomized trial by Fournier et al,^19^ patients receiving CABG using FFR for severity evaluation in at least one vessel were compared to patients receiving solely angiography-guided CABG. TVR was similar between the 2 groups. However, the revascularization rate was numerically higher in the FFR-guided group compared to the angiography-guided group, similar to the present study. This difference was also mainly seen between 12 and 36 months, becoming similar after 5 years. Interestingly, none of the TVR events in the FFR-guided group occurred in deferred lesions, in contrast to the present study, where more than one-fifth occurred in a deferred lesion.

FFR-guided deferral of PCI in angiographic 3-vessel disease has also been found to be associated with increased new revascularization compared to angiography-guided CABG, as seen in the long-term follow-up of the FAME 3 (Fractional Flow Reserve Versus Angiography for Multivessel Evaluation 3) trial.^21^ However, this difference was first seen after 5 years of follow-up, with no statistical difference observed after 1 year,^22^ signaling that the increased revascularization rate in FFR-guided PCI is more related to the durability of PCI rather than the safety of FFR-deferral. Although there was a numerically higher revascularization rate already after 1 year, this difference was not statistically significant. This differs from the results of the present study, where the increased revascularization rate was mainly driven by increased revascularization during the first year, with no difference in long-term outcomes after 1 year. The differing patterns of disease progression in FFR-guided deferral of PCI and CABG suggest differing mechanisms behind the increase in revascularization.

One potential reason for the lack of benefit, and potential reduced efficacy, associated with FFR-guided CABG, but not with FFR-guided PCI, is the differing consequences of revascularization failure. The failure of PCI, although rare, results in narrowing or acute occlusion of the native vessels, by either in-stent restenosis or stent thrombosis, associated with adverse long-term outcomes. In contrast, the failure of CABG may be more subtle. In the setting of borderline stenosis, graft failure may be clinically silent and graft occlusion does not necessarily impair native blood flow. Therefore, the risk associated with grafting functionally borderline stenoses may be lower than the long-term risk of PCI, and grafting deferral may therefore be less important than stent deferral. Thus, the benefits of complete anatomical revascularization might outweigh the relatively small risk of grafting a nonfunctionally significant stenosis, while the opposite could be true for PCI.

Although FFR-guided CABG resulted in fewer grafted vessels, graft failure was still the most frequent reason for revascularization in the FFR-guided group, having even numerically more frequent graft failure revascularization than the angiography-guided group. The potential mechanism behind the increased graft failure revascularization is unclear. While the reduction in grafted vessels is an intended effect of the FFR guidance, this also may result in less complete anatomical revascularization. The consequences of graft failure in patients with less complete anatomical revascularization might be greater, as less of the coronary tree is anatomically revascularized and thereby more susceptible to impaired blood flow following graft failure. However, further research into the effects of FFR-guided CABG on graft failure revascularization and the consequence of less complete anatomical revascularization is needed.

While the increased revascularization in the present study appears, in part, to be related to the deferred lesions, accounting for one-fifth of revascularizations, the mechanism behind a midterm FFR reduction and subsequent increased revascularization is still uncertain. Coronary blood flow is altered after CABG, with flow being reversed in some segments of the coronary artery, resulting in changes in wall shear stress that may promote native disease progression in grafted arteries.^23^ These hemodynamic changes created by grafting might also influence nearby vessels and could increase flow, which has also been shown to reduce FFR.^24^ Furthermore, the increased systemic inflammation and reduced daily activity caused by the CABG operation might give rise to a transient increase in the atherosclerotic process during the first year following CABG. Finally, the observed reduction might represent a regression to the mean phenomenon. However, this would not explain the observed increased revascularization rate seen in the present study.

The difference in revascularization between the 2 groups occurred primarily in relation to the 6-month angiographic follow-up. While angiographic follow-up is often associated with study-induced revascularization, both the FFR-guided and angiography-guided groups underwent angiographic follow-up. It is possible that the mandatory FFR evaluation in the FFR-guided group could bias operators to perform revascularization. However, all revascularizations of deferred lesions at 6-month follow-up were ischemia-driven, and patients with functionally significant FFR values but without symptoms or with diffuse disease were not revascularized, suggesting that the results of the present study pose a real clinical challenge.

No differences were observed between FFR-guided and angiography-guided CABG with regard to MI, stroke or death, MI or death, nor in a combined safety endpoint. These findings suggest that, despite an increased revascularization rate, FFR-guided CABG remains a safe strategy. Similar results were reported in the GRAFFITI trial, where no differences with regard to all-cause mortality, spontaneous MI, or stroke were observed. Similarly, in Fournier et al,^19^ there were no differences with regard to MI and actually lower all-cause mortality. Although, the mortality benefits were no longer observed when matching the FFR-guided patients to the angiography-guided patients. However, it should be emphasized that the results of this study should be interpreted with caution, as the study was not powered for clinical endpoints, nor for assessment of safety. Furthermore, the study was also conducted in a population in which three-quarters of patients presented with stable symptoms. Therefore, the risks of MI, stroke, and death are generally low, and larger studies are needed to further evaluate the safety of FFR-guided CABG with regard to hard clinical outcomes.

### Study Limitations

While all repeat revascularizations were symptom-driven, the main difference in the Kaplan-Meier curve for the 2 groups was seen around the 6-month follow-up, and the potential influence of the mandated coronary angiogram with repeat FFR represents an important limitation of the study. The present study was conducted unblinded by both the treating physician and the operator. This introduced the potential for bias, especially considering that the difference was observed in new revascularization, which can greatly be influenced by bias due to the inherent subjectiveness in assessment and interpretation of symptoms of ischemia. This is further increased by the mandatory invasive follow-up and FFR evaluation, which may have influenced both the treating operator and patients in interpreting the symptoms. The differences in revascularization observed in the present study may therefore have been smaller if no invasive follow-up was conducted and may partly explain the relatively high event rates observed. This is also a potential explanation for the violation of the proportional hazards assumption seen in the revascularization rate during the first year. This is further supported by the sensitivity analysis excluding the events at 6-month follow-up, where the differences in MACCE and revascularization diminished and were no longer statistically significant. However, these results should be interpreted with caution given the small sample size and low number of events observed in the study. In addition, exclusion of the revascularization events detected at 6-month follow-up may itself introduce bias. The additional revascularization in the FFR-guided group at 6-month follow-up may have altered the number and characteristics (native vs grafted vs stented) of lesions and thereby influence the risk of subsequent revascularization during the remainder of follow-up. Furthermore, since the extended follow-up was based on the electronic patient journal, no objective symptom scores were available, and symptom-driven revascularization was based on physician assessment of symptoms, which may have been biased by the unblinded nature of the study. However, all deferred lesion revascularizations at 6-month follow-up were ischemia-driven based on invasive FFR and patients’ symptoms. Although the unblinded nature of the study creates bias in the interpretation of the results, it also mimics daily clinical practice. In addition to the limitations discussed above, the main limitation of the study is the number of included patients. The FARGO trial was ended prematurely due to a low inclusion rate; however, it still is one of the largest randomized controlled trials of FFR-guided CABG vs angiography-guided CABG to date. Another limitation is that in 12% of patients, the graft plan could not be followed due to small-caliber arteries or lack of grafting material. This might influence the results of the study but also reflects daily clinical practice. Furthermore, since the present study was an extended follow-up of the original FARGO trial, only information on clinical endpoints was collected, and information regarding risk factor control and medical therapy was not collected, limiting analysis of the potential influence of risk factors on FFR deferral. Finally, this study was not powered for clinical outcomes and should therefore only be considered hypothesis-generating.

## Conclusions

FFR-guided CABG was associated with a significantly higher rate of MACCE after CABG compared to angiography-guided CABG, driven by more revascularization within the first year.

Perspectives **COMPETENCY IN MEDICAL KNOWLEDGE:** While FFR-guided PCI has demonstrated clinical benefit, the role of FFR to guide CABG remains uncertain. FFR-guided CABG may result in a greater risk of MACCE, driven primarily by an increased rate of repeat revascularization compared to angiography-guided CABG. While no difference is observed in all-cause mortality, MI, or stroke.

**TRANSLATIONAL OUTLOOK:** Larger randomized clinical trials, powered for clinical endpoints, are warranted to define the long-term clinical impact of FFR-guided CABG.

## Funding support and author disclosures

Dr Jensen has received unrestricted research grants to her institution from Meril, Biotronik, Biosensors, and OrbusNeich. Dr Eftekhari has received an institutional grant from Biosensors and OrbusNeich. Dr Christiansen has received a research grants from Abbott. Dr Modrau has received an unrelated grant from Novo Nordisk Foundation (#0083480). All other authors have reported that they have no relationships relevant to the contents of this paper to disclose.

## Appendix

For a supplemental table, please see the online version of this paper.
