# Supplementary material for: Long-Term Clinical Outcomes After Physiology-Guided vs Angiography-Guided Coronary Artery Bypass Grafting: From the FARGO Trial
Source: JACC Adv. 2026 Sep 22;5(11):103332. doi: 10.1016/j.jacadv.2026.103332 (PMC13685978; doi:10.1016/j.jacadv.2026.103332)
Supplement: Supplementary file 1 [file mmc1.docx]

**Supplementary Appendix**

Table of contents

Supplementary Table S1……………………………………………………………………………...2

| Supplementary Table S1 – Fine-Gray competing-risk sensitivity analyses | | |
| --- | --- | --- |
|  | sHR (95% Confidence interval) | p-value |
| Any new revascularization | 3.47 (1.36-8.87) | 0.009 |
| Myocardial infarction | 2.01 (0.52-7.78) | 0.31 |
| Stroke | 1.08 (0.35-3.37) | 0.89 |
| Fine-Gray competing-risk regression with death as competing event for FFR-guided vs angiography-guided. Center, sex and presence of diabetes included as covariates. sHR: Subdistribution hazard ratio, CI: Confidence interval | | |
